# Novel evidence of obesity paradox in esophageal adenocarcinoma: perspective on genes that uncouple adiposity from dismal outcomes

**DOI:** 10.7150/jca.65138

**Published:** 2022-01-01

**Authors:** Lei Zhu, Fugui Yang, Lin Dong, Guangxue Wang, Qinchuan Li, Chunlong Zhong

**Affiliations:** 1 Department of Neurosurgery, Shanghai East Hospital, School of Medicine, Tongji University, Shanghai 200120, China.; 2 Department of Thoracic Surgery, Shanghai East Hospital, School of Medicine, Tongji University, Shanghai 200120, China.; 3 Research Center for Translational Medicine, Shanghai East Hospital, School of Medicine, Tongji University, Shanghai 200120, China.

**Keywords:** esophageal adenocarcinoma, obesity paradox, protective effect, prognosis, bioinformatics analysis

## Abstract

**Background:** Obesity is a strong risk factor for esophageal adenocarcinoma (EAC). Nevertheless, not all the patients with EAC are obesity, and a substantial proportion of obesity patients don't suffer from poor prognoses. The mechanisms behind the “obesity paradox” that uncouple obesity from dismal outcomes in EAC are unclear. This study aimed to explore the “obesity-guarding” genes (OGG) profiles and their prognostic values in patients with EAC.

**Methods:** Gene expression data and clinical information of patients with EAC were downloaded from The Cancer Genome Atlas (TCGA) database. Enrichment analysis was used to explore the OGG functions and pathways. Cox regression analysis and nomogram model were performed to investigate the OGG prognostic values for overall survival (OS). In addition, relations between OGG and immune cells were assessed by the “CIBERSORT” algorithm and the Tumor IMmune Estimation Resource (TIMER) tool. Finally, the results were experimentally validated in real-world study.

**Results:** A total of 69 OGG were retrieved, and 17 significantly differentially expressed genes (SDEG) were identified between normal and EAC tissues. Enrichment analysis showed the OGG were enriched in the mitochondrion-related and various receptor pathways. Univariate Cox regression results showed that the MCM6, ATXN2 and CSK were significantly associated with OS (P=0.036, 0.039, 0.046, respectively). Multivariate Cox regression analysis showed MCM6 and CSK were independent prognostic genes for OS (P=0.025, 0.041, respectively). Nomogram demonstrated that the OGG had good predictive abilities for the 1-, 2-, and 3-year OS. Immunity analysis demonstrated that OGG were significantly associated with immune cells (P <0.05). In addition, clinical correlation analysis revealed that the OGG had significant relations with clinical parameters (P <0.05). The experiment results confirmed that the SDEG were significantly different between normal and EAC tissues (P <0.05).

**Conclusions:** We identified the OGG expression profiles that may uncouple obesity from poor survival in patients with EAC. They have prognostic values in predicting patients' OS, and may be exploited for prognostic biomarkers.

## Introduction

Esophageal cancer is the eighth most common malignancy worldwide, and the esophageal adenocarcinoma (EAC) prevails in the Western countries as the prominent pathological type 1, 2. EAC is characterized by high incidence and dismal prognosis. The most recent estimate by the Surveillance Epidemiology and End Results (SEER) analysis revealed that EAC incidence surged from 0.4/100,000 person-years to 3.5/100,000 person-years in the last four decades, and the 5-year survival rate is less than 20% 3, 4. These occurred against the background that obesity prevalence rose from approximately 10% in 1975 to 30% in 2016 globally, which paralleled with the increasing incidence of EAC 5. Since the first case-control study discovered the possible association between obesity and EAC, numerous studies concluded a consensus that obesity was a risk factor for EAC development and prognosis 6, 7, 8. Recently, an umbrella review based on 204 meta-analyses showed strong evidence that increased body mass index (BMI) was associated with a higher risk of developing EAC 9. Although the underlying mechanisms of how obesity contributed to the drastic increase and poor outcomes of EAC are largely unknown, several reasons tentatively accounting for the causality are being put forward, including the reprogrammed tumor microenvironment (TME) and compromised immunosurveillance 10.

However, on the contrary, there are also many unexpected results 11, 12. For example, obesity is more prevalent in African Americans and East countries, yet the EAC incidence is pretty low. Furthermore, not all obesity is associated with poor prognoses. Previously, a meta-analysis reported that EAC patients with overweight or obesity didn't have worse survival compared with those of normal weight 13. Later, Zhang SS et al provided reliable evidence that EAC patients with higher BMI had significantly increased overall survival (OS) 14. A review across different literatures by Wong JY et al. summarized that obesity didn't pose significant impact on esophageal cancer patients' outcomes 12. In fact, it's estimated that 30-50% overweight and obesity adults had healthy profiles and were away from metabolic disturbances 15. Instead, more than 20% normal-weight adults have abnormal profiles similar to the obesity 15. Therefore, it's reasonable to speculate obesity per se may not be culprit for EAC. The wide applications of gene sequencing technology have been revealing the mechanisms behind the “obesity paradox” phenomenon gradually. The latest study by Huang LO and their cooperative team through the largest public genome-wide association studies (GWAS) database and the UK Biobank population based on more than 300,000 individuals, discovered and validated 62 loci with corresponding 69 genes expressed in adipose tissues (a locus affects one or more genes nearest to it) 16. These genes influence adipocyte tissue functions and protect the obese individuals from detrimental complications, which may partly explain the “obesity paradox” in obese EAC patients with better prognoses. This new finding is important in conveying healthy public concepts. For instance, it's not the lean people were healthier and didn't have necessary to keep fit. In addition, it needs to be emphasized that this study shed light on novel mechanisms to uncover “the obesity paradox”, and these genes may serve as an innovative strategy for clinical management, not a promotion to encourage people to gain overweight.

To identify the 69 genes roles and gain further insights into the possible mechanisms uncouple obesity from worse prognoses, we performed, to our best known, the first comprehensive analysis of the 69 genes expression profiles in patients with EAC. We found the genes signatures were significantly different between normal and EAC tissues, and they have close relationships with immunity. Moreover, the genes could serve as prognostic biomarkers in predicting OS. Finally, we experimentally validated the results using the clinical specimens in our hospital.

## Materials and methods

### Data acquisition and patients' clinical information

The gene expression data and corresponding clinical information in patients with EAC were downloaded from The Cancer Genome Atlas (TCGA) database (https://portal.gdc.cancer.gov/).

The inclusion criteria were as follows: (1) patients with complete gene expression profiles; (2) patients with complete survival time and survival status; (3) patients with complete pathological grades. Exclusion criteria: (1) esophageal squamous cell carcinoma; (2) OGG expression levels not available; (3) without complete clinical and pathological information; (4) samples number less than 50; (5) lost to follow up. The RNA sequences from TCGA were calculated through fragments per kilobase of per million (FPKM) algorithms. The significantly differentially expressed genes (SDEG) between normal and EAC tissues were identified by the “limma” R package with the false discovery rate (FDR) < 0.05. Patients' clinical information included gender, tumor grade, TNM stage, survival status and survival time. Among them, tumor grade represents the degree of abnormality of cancer cells, and can be classified into G1, G2, G3 and G4. TNM stage contains three parameters, in which T refers to the size or contiguous extension of the primary tumor (T), N refers to the stage of cancer based on the nodes present (N stage), M represents the defined absence or presence of distant spread or metastases (M). Survival time represents the interval from the date of last follow up to the date of initial pathologic diagnosis, and usually refers to OS.

The 69 “obesity-guarding” genes (OGG) were obtained from the previous study, which was used to describe a set of adiposity-protection loci and genes without accompanying cardiometabolic comorbidities 16. Herein, OGG refer to genes that uncouple excess adiposity from comorbidities and protect the obese from poor outcomes in patients with EAC. The characteristics of the 69 OGG were provided in Supplementary Table S1. The SDEG interactions analysis was conducted using the “corrplot” and “igraph” packages in R software.

### Enrichment analysis and semantic similarities

Gene Ontology (GO) enrichment analysis included the biological process (BP), cellular component (CC) and molecular function (MF), and was conducted by R software using the “clusterProfiler” R package. Kyoto Encyclopedia of Genes and Genomes (KEGG) was also performed by the same tool. The criteria were set as follows: |log2FC| >1 and FDR < 0.05.

Functional similarity refers to the geometric mean of their semantic similarities in GO enrichment analysis, which could be used for the purpose of assessing the intimacy and relationship between each gene and its partners by evaluating function and location. Semantic similarities among functional OGG were measured through the “GOSemSim” R package 17.

### Signatures for survival prediction

Univariate and multivariate Cox regressions were performed to evaluate the prognostic values of OGG for OS. Then, independent prognostic genes selected from multivariate Cox regression were used to synthesize the risk score, which was calculated by the following formula: risk score = 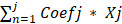, with* Coef j* representing the coefficient and *Xj* representing the relative expression levels of each prognostic gene standardized by z-score. Patients were divided into high- and low-risk groups according to the median of the risk score.

Next, we combined the patients' clinical information with risk score, and examined the relationships between clinical information and OS. In addition, to assess the feasibility of the survival predictive model, nomogram was developed by the “regplot” and “rms” R packages. Its predictive ability was assessed by the calibration curve. Furthermore, the receiver operating characteristic (ROC) curve was also built to evaluate the predictive performance by the “pROC” R package. The area under curve (AUC) of the ROC ranges from 0.5 to 1, with 1 indicating perfect predictive ability and 0.5 indicating no predictive ability.

### Assessing the OGG effects on immune cells

We first calculated the infiltration immune cells contents in each EAC sample through the “CIBERSORT” tool 18. CIBERSORT is an analytical tool where the algorithm was run using the LM22 (a leukocyte gene signature matrix termed by Newman AM) signature at 1000 permutations 14, which was available on the website (https://cibersort.stanford.edu/index.php). Next, single-sample gene set enrichment analysis (ssGSEA) was used to quantify the immune cells by the“GSVA” R package 19. Then, we evaluated the effects of the OGG on immune cells using linear regression. Finally, the relations between OGG and immune cells were validated in the TIMER database (https://cistrome.shinyapps.io/timer/).

### Experimental validation from real-world study

To verify the results, we conducted the polymerase chain reaction (PCR) to validate the SDEG expression levels in Shanghai East Hospital, School of Medicine, Tongji University after the approval of the Internal Review Board. Fifteen clinical specimens were collected from patients with EAC who underwent esophagectomy in our institution from 2019 January to 2020 June. Fifteen normal esophageal mucosal tissues were set as control.

Total RNA from EAC and normal tissues were extracted through Trizol reagent (3 ml/100 mg, Sigma-Aldrich). Chloroform (0.5 ml/1 ml trizol, Sigma-Aldrich) and isopropanol (0.5 ml/1 ml trizol, Sigma-Aldrich) were added and centrifuged. Complementary DNA (cDNA) was synthesized from 1 μg of total RNA using a PrimeScript® RT reagent Kit with genomic DNA (gDNA) Eraser (Takara, Dalian, China). Reverse Transcription PCR (RT-PCR) was implemented in the 7500 Fast Real-Time PCR System (Applied Biosystems, USA). The endogenous glyceraldehyde-3-phosphate dehydrogenase (GAPDH) and actin served as the internal control. The gene primers were synthesized by Sangon Biotech (Shanghai) Co., Ltd, China, and the primers were listed in Supplementary Table S2.

### Statistical analysis

Gene expression differences were calculated by the student's t-test between the normal and EAC tissues. Gene interactions analyses were done using the “corrplot” and “igraph” R packages. Univariate and multivariate Cox regression analyses were performed to identify the prognostic predictors for OS. Log-rank test and Kaplan-Meier curve were used to compare and visualize the survival differences between high- and low-risk groups. Mann-Whitney U test was used to compared the immune score, immune cell infiltrations and immune signatures. Spearman correlation analysis was used to evaluate the interactions. All the statistical analyses were completed by the R software (version 4.0.3). P-value < 0.05 was set as statistically significant in the present study.

## Results

### Gene expression profiles and patients' characteristics

**Figure 1:**
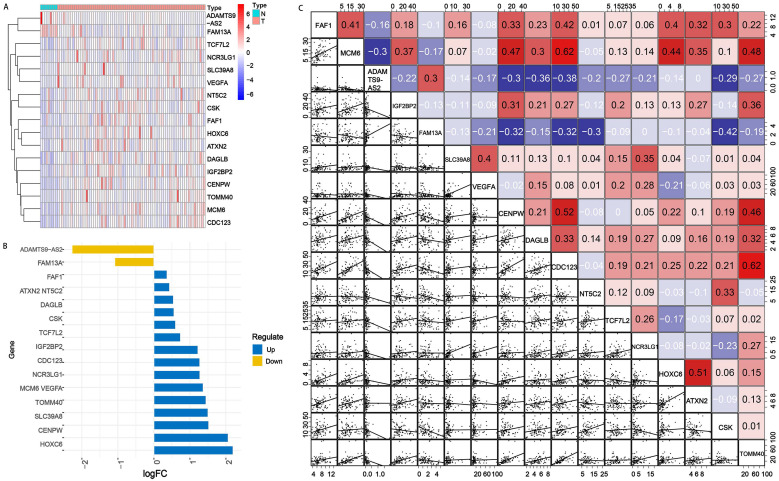
** SDEG expression levels in normal and EAC tissues. A.** Heatmap of SDEG. The blue represented low expression in EAC tissues compared with normal tissues, while the red represented high expression. **B.** Bar plot of the SDEG. Among the 17 SDEG, 15 genes were upregulated and 2 genes were downregulated. **C.** Gene interaction analysis of 17 SDEG. Red boxes represented the positive correlation while negative in blue. N: normal tissues; T: EAC tissues; logFC: log fold change.

There were 87 samples with corresponding genes expression data downloaded from TCGA database, including 9 normal and 78 EAC samples. Among the 69 OGG, 24.64% (17/69) were SDEG between EAC and normal tissues. ADAMTS9-AS2 and FAM13A were downregulated and the other 15 genes were upregulated significantly in EAC tissues compared with normal tissues according to an FDR <0.05 (Figure 1A and 1B). The 17 SDEG expression profiles and 69 OGG raw data were provided in Table 1 and Supplementary Table S3.

Next, we investigated the gene interactions, and the result showed MCM6 had the strongest positive correlation (r=0.62), CSK had the strongest negative correlation with FAM13A (Figure 1C).

### Enrichment analysis and gene interaction network

**Figure 2:**
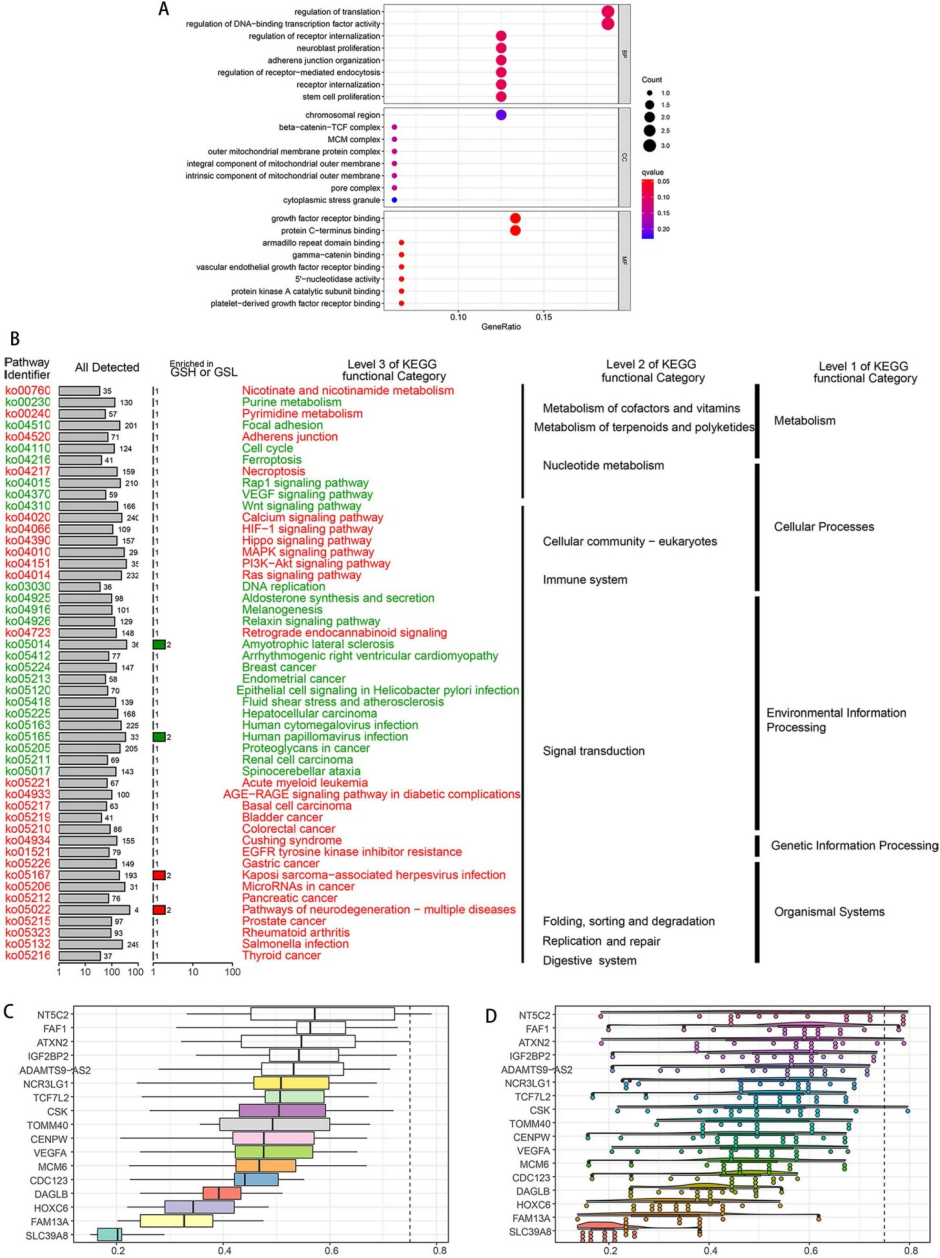
** Enrichment analyses of SDEG. A.** GO enrichment analysis, including BP, CC and MF. GO results showed the SDEG were enriched in receptor internalization pathway, mitochondrion-related activities and receptor binding pathways. KEGG result displayed that SDEG had significant relationships with metabolism, cellular processes and genetic information processing. **C.** Summary of OGG similarities. The boxes indicated the middle 50% of the similarities; and the upper and lower boundaries show the 75^th^ and 25^th^ percentile. **D.** Raincloud plots of OGG. Data were shown as the mean and standard error. Each dot represented the single gene. The dashed line represents the cutoff value (0.75).

To explore potential functions of the SDEG, we performed the GO and KEGG enrichment analysis. The GO results showed the SDEG were enriched in receptor regulation and mitochondria-related pathways. BP results demonstrated the genes were strongly associated with receptor internalization regulation, neuroblast proliferation and adherens junction. CC results demonstrated the genes were evidently correlated with mitochondria-related pathways. MF results displayed the genes had close relations with growth factor receptor, armadillo repeat domain and gamma-catenin binding (Figure 2A). KEGG results clearly showed the genes were enriched in the metabolism, cellular processes, genetic information pathways (Figure 2B).

According to the semantic similarities, we ranked the genes by average functional similarities between OGG and their partners, with the cut-off value 0.75 (Figure 2C). NT5C2, FAF1 and ATXN2 had the strong similarities, and had weak correlations with SLC39A8. The distributions of OGG similarities were demonstrated in Figure 2D.

### Prognostic genes and independent risk factors

**Figure 3:**
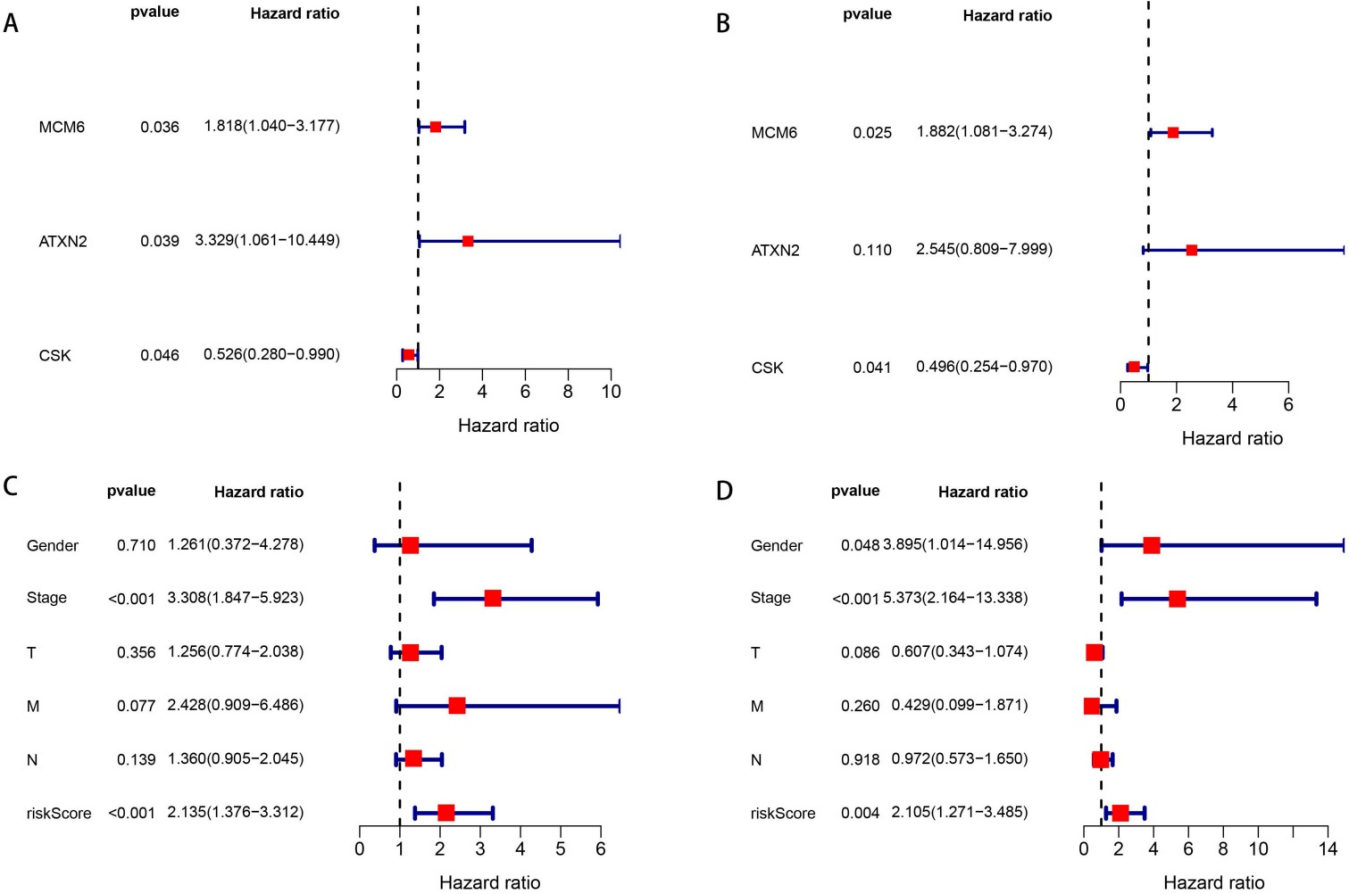
** Forest plots of univariate and multivariate Cox regression analyses of OS. A.** Univariate Cox regression analysis of SDEG for OS. **B.** Multivariate Cox regression analysis of SDEG for OS. **C.** Univariate Cox regression analysis of clinical information and risk score for OS. **D.** Multivariate Cox regression analysis of clinical information and risk score for OS. The contents in the brackets represent the 95% confidence interval.

We enrolled the 69 OGG into the univariate Cox regression analysis to identify prognostic genes. The results showed MCM6, ATXN2 and CSK were significantly associated with OS in patients with EAC (P=0.036, 0.039, 0.046, respectively) (Figure 3A). Next, multivariate Cox regression analysis showed MCM6 (HR=1.882, P=0.025) and CSK (HR=0.496, P=0.041) were independent prognostic genes, in which the MCM6 was the risk gene (HR>1) and CSK was the protective gene (HR<1) (Figure 3B).

Then, according to the median of the risk score (risk score = 0.632 * expression level of MCM6 + -0.701 * expression level of CSK), patients with EAC were classified into high- and low-risk groups. We combined the risk score with patients' clinical information to assess their prognostic values for OS. In the univariate Cox analysis, we found that tumor stage and risk score were significantly associated with OS (all P <0.001) (Figure 3C). The multivariate Cox analysis demonstrated patients' gender (HR=3.895, P=0.048), tumor stage (HR=5.373, P<0.001) and risk score (HR=2.105, P=0.004) were independent risk factors for OS (Figure 3D).

### Construction of prognostic hazard curves and ROC model

**Figure 4:**
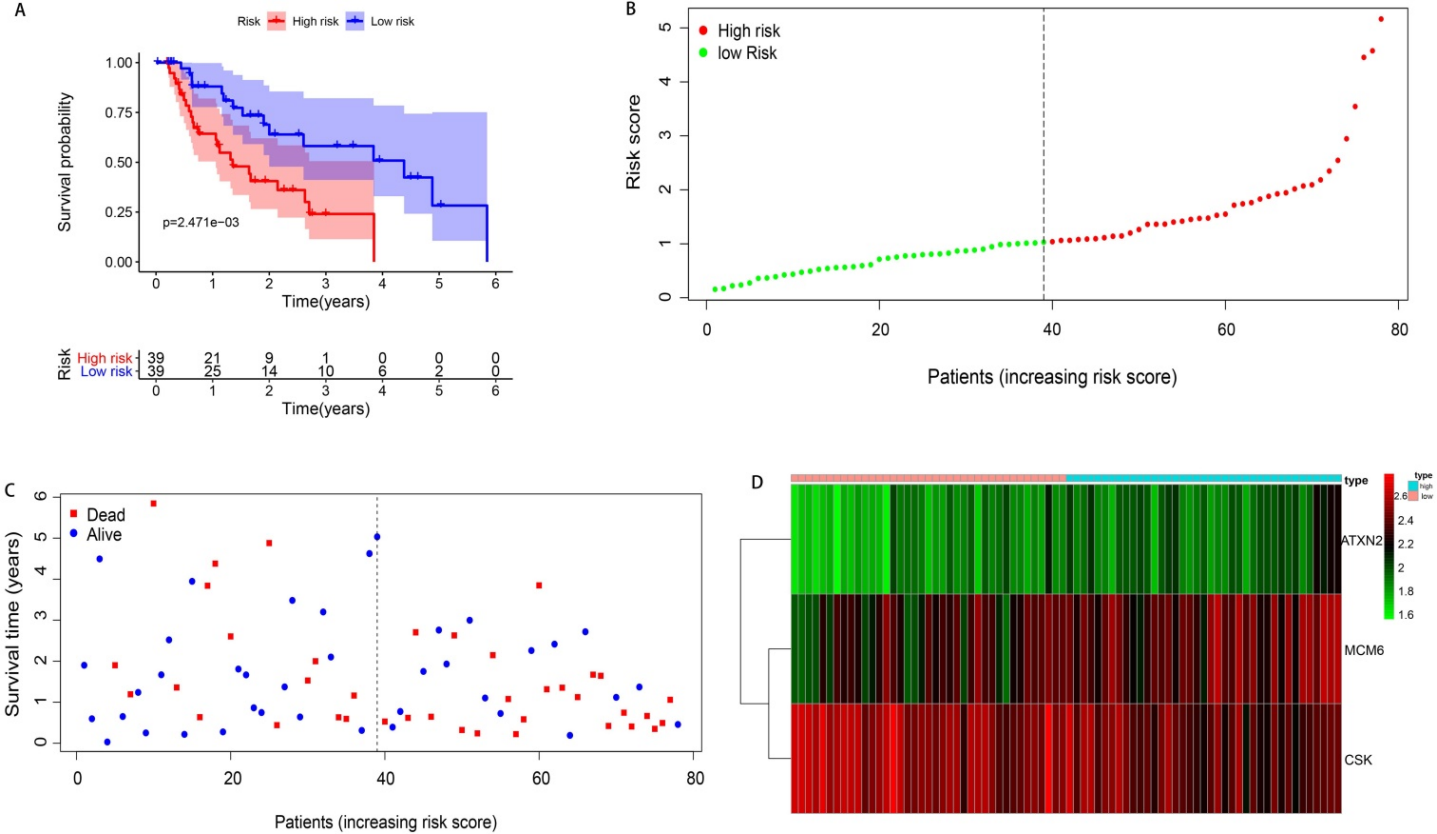
** Kaplan-Meier curve survival analysis and prognostic hazard curves. A.** Kaplan-Meier curve demonstrated the high-risk group patients had significant worse survival than those in the low-risk group. **B.** The distribution and median value of the risk score. Green dots represented the low-risk patients and red dots represented the high-risk patients in EAC. **C.** The distributions of OS in EAC. The blue dots represented the alive patients, while the dead in red. **D.** Heatmap of prognostic genes in high- and low-risk groups. ATXN2 and MCM6 were at higher expression level in high-risk group, and CSK at lower expression level.

According to the median of the risk score, 78 patients with EAC were divided into high- and low-risk groups (n=39 respectively). The survival time of patients in the low-risk group was significantly longer than those in the high-risk group (median time =1.529 years VS 1.077 years, P=0.002). The Kaplan-Meier curve was shown in Figure 4A. The patients' death risk increased, and survival time decreased with the increase of the risk score (Figure 4B, C). In addition, the risk heatmap was developed and we can clearly see the MCM6 was overexpressed in the high-risk group, implying it was an oncogene. However, the CSK was downregulated in the high-risk group, indicating it was a protective gene (Figure 4D).

**Figure 5:**
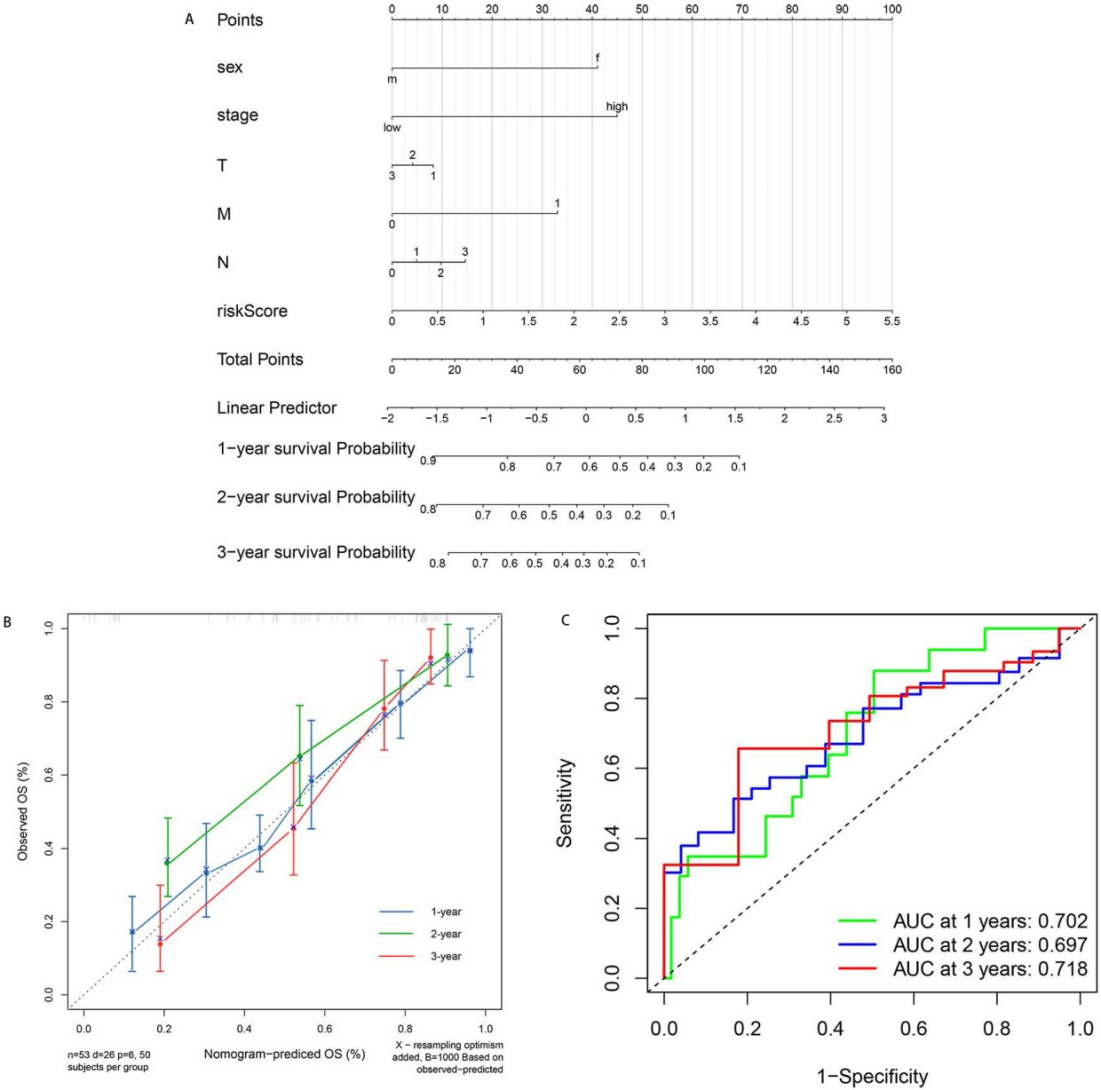
** Nomogram for predicting the patients' survival based on the risk score and clinical information. A.** Nomogram plot for predicting 1-, 2- and 3-year OS. **B.** Calibration curves of nomogram based on risk score in terms of agreement between predicted and observed 1-, 2- and 3-year survival. **C.** ROC model for the prognostic performance of the risk score. The area under curve (AUC) ranges from 0.5 to 1.0, and the predictive abilities for 1-, 2- and 3-year survival rate were 0.702, 0.697 and 0.719 respectively.

Furthermore, to provide an accurate method to predict the OS, we established the nomogram based on the risk score and clinical information (Figure 5A). The calibration curves for 1-, 2- and 3-year demonstrated excellent consistency with the standard curve, suggesting superior performance (Figure 5B). In addition, we built the ROC model to test the predictive accuracy and calculate the AUC. As is shown in Figure 5C, our ROC model achieved the AUC of 0.702, 0.697 and 0.718 for 1-, 2-, 3-year survival rates for OS. The performance of ROC model was excellent and exhibited feasibility.

### Correlation of OGG with immune cells

By applying the “CIBERSORT” algorithm to gene expression, we firstly obtained the relative expression levels of 22 immune cells in EAC sample. The compositions and expressions of 22 immune cells in each EAC sample were provided in Supplementary Table S4. Then, we investigated the effects of SDEG on immune cells by the linear regression analysis. The results demonstrated that 11 genes, including the ADAMTS9-AS2, CDC123, CENPW, CSK, DAGLB, FAM13A, HOXC6, SLC39A8, TCF7L2, TOMM40 and VEGFA had significant effects on the immune cells compositions (all the P <0.05). The detailed correlations were presented in Table 2.

**Figure 6:**
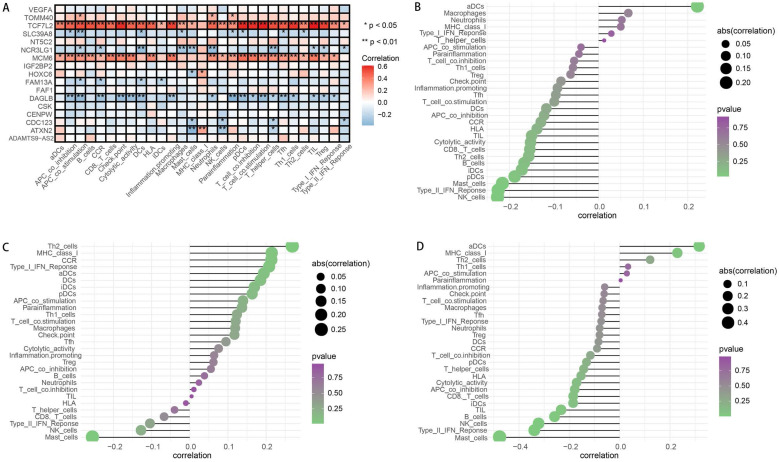
** Analysis of immune cells and immune functions. A.** Heatmap depicting the correlation between 17 SDEG and the ssGSEA scores of 29 immune signatures. **B-D.** Correlation between risk score (B), ATXN2 (C), MCM6 (D) and immune cells. Spearman correlation analysis was used to evaluate the relations with P<0.05.

**Figure 7:**
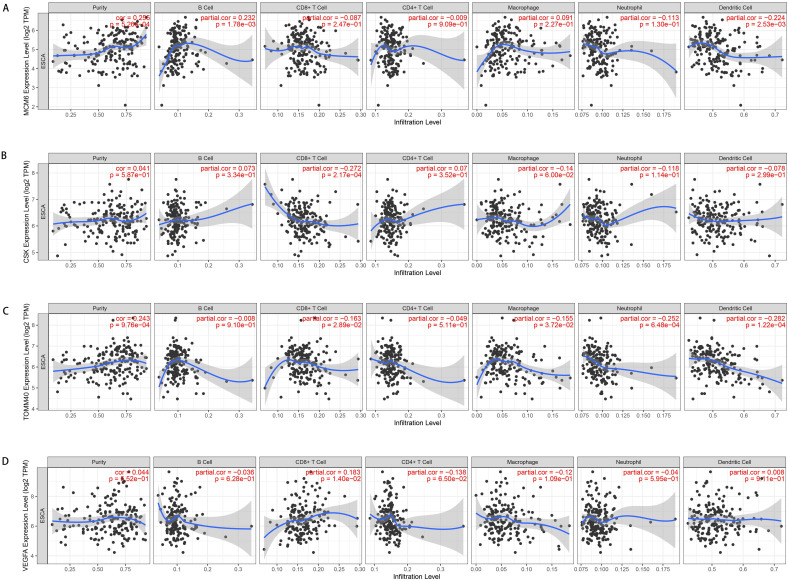
** Representative results of relations between OGG and immune cells in TIMER database. A.** MCM6 expression level and immune cells in esophageal cancer.** B.** CSK and immune cells; **C.** GLS2 and immune cells; **D.** VEGFA and immune cells. TPM: transcripts per kilobase million.

Subsequently, we assessed the effects of OGG on immune cells and immune functions through the “ssGSEA”. Consistent with the CIBERSORT results, the OGG were significantly associated with immune cells and their functions (P <0.05). Twenty-nine immune signatures were shown in Figure 6A. We further explored the associations between the prognostic genes and immune cells, and we found that the risk score, ATXN2 and MCM6 were positively or negatively correlated with T cells and mast cells, et al. (P <0.05) (Figure 6B-D). Finally, to validate the immune cells and their relations with SDEG, the TIMER database was employed. In line with the findings, the immune cells were significantly correlated with SDEG (Figure 7).

### Association of the OGG and clinical parameters

**Figure 8:**
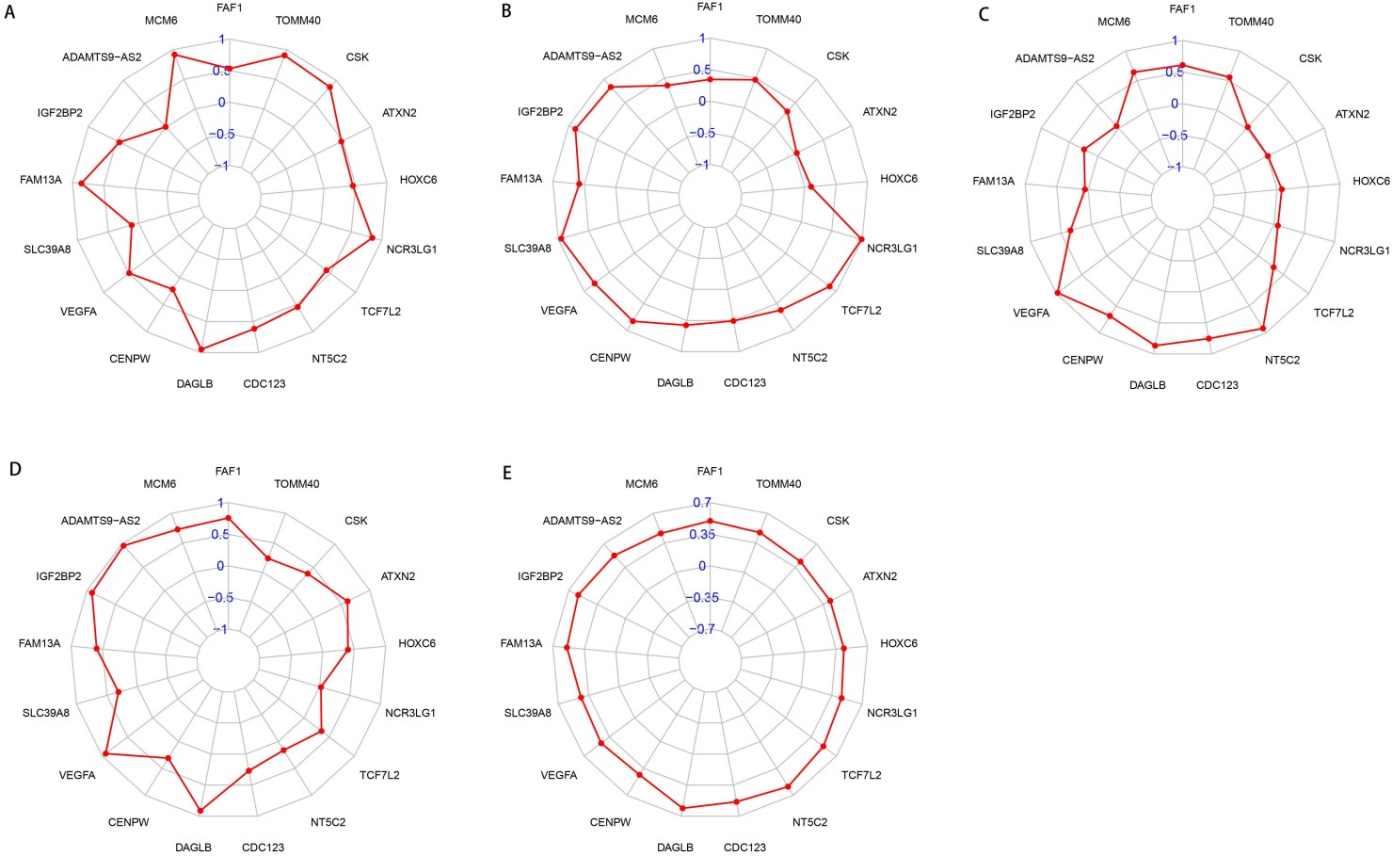
** Radar maps of SDEG and their correlations with clinical parameters. A.** SDEG expression levels and their correlations with gender in patients with EAC. **B.** SDEG and tumor stage. **C.** SDEG and primary tumor condition. **D.** SDEG and patients survival status. E: SDEG and survival time. P < 0.05 was considered statistically significant.

To better understand the roles of OGG in EAC, we analyzed the relationships between OGG and clinical parameters (gender, tumor stage, TNM, survival time and survival status). Our findings showed that NCR3LG1 was negatively associated with survival status (R=-2.335, P=0.024), ADAMTS9-AS2 negatively associated with gender (R=-3.066, P=0.005), ATXN2 negatively associated with tumor stage (R=-2.264, P=0.030), ATXN2 and CSK positively correlated with tumor T stage (primary tumor condition) (R=10.152, P=0.006; R=7.068, P=0.029, respectively). The details were visualized in Figure 8.

### Experimental validation

**Figure 9:**
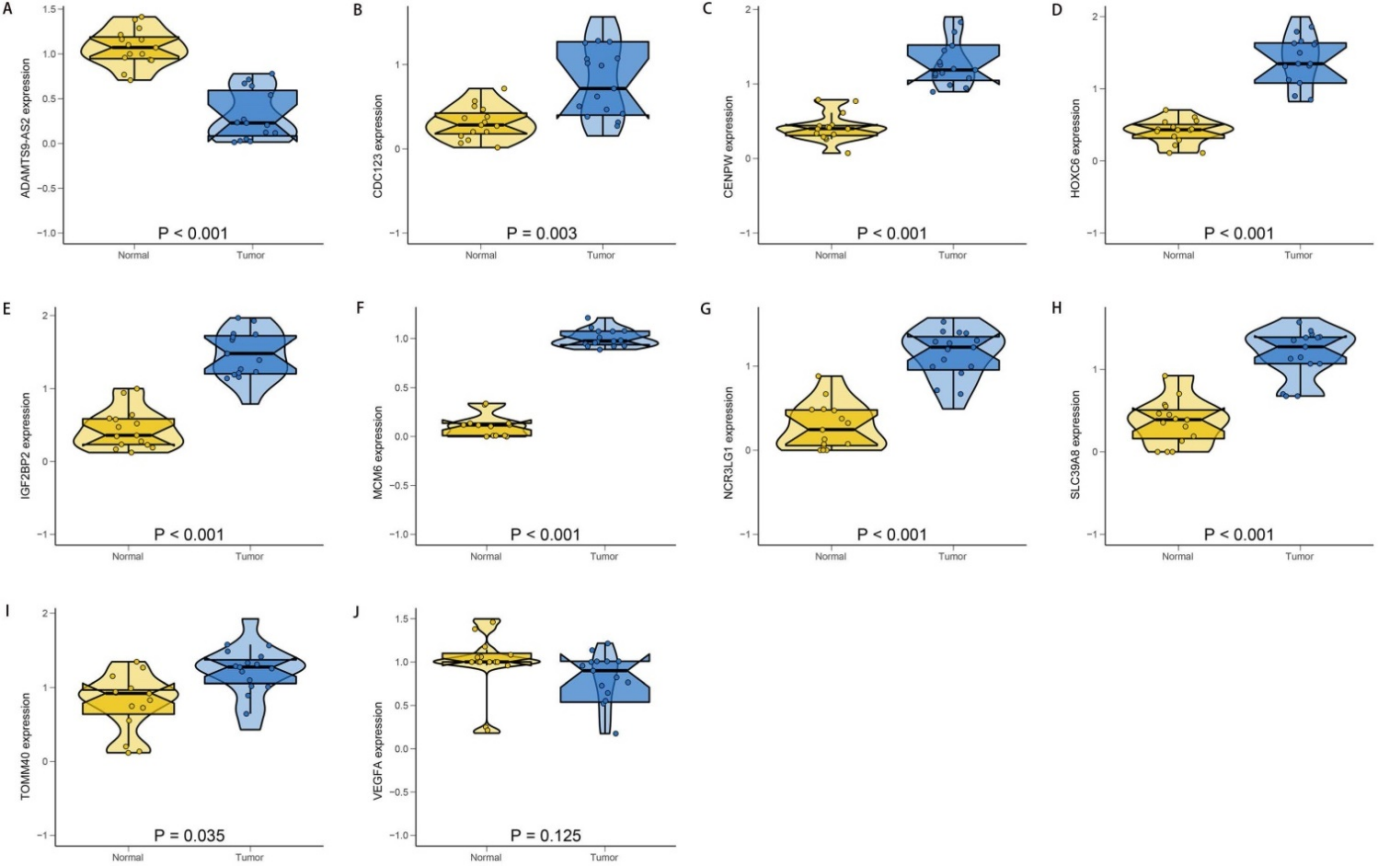
** The relative expression levels of the top ten SDEG.** ADAMTS9-AS2 **(A)** was significantly down-regulated in EAC tissues. There were no significant differences in the expression of VEGFA **(J)** between normal and EAC tissues. While, the CDC123 **(B)**, CENPW **(C)**, HOXC6 **(D)**, IGF2BP2 **(E)**, MCM6 **(F)**, NCR3LG1 **(G)**, SLC39A8 **(H)** and TOMM40 **(I)** were significantly up-regulated in EAC tissues compared with the normal tissues. N: normal; T: EAC.

To verify the accuracy and reliability of the above findings from TCGA database, we selected the top ten most significantly different genes and we conducted the PCR to validate the OGG expression levels in clinical specimens. Consistent with the bioinformatics results in our study, CDC123, CENPW, HOXC6, IGF2BP2, MCM6, NCR3LG1, SLC39A8 and TOMM40 were upregulated in the EAC tissues compared with the normal esophageal mucosa tissues. The ADAMTS9-AS2 was significantly downregulated in EAC tissues (Figure 9). The gene expression differences suggested that the “obesity-guarding” genes played important roles in EAC.

## Discussion

Different tumors display diverse epidemiological characteristics, and express heterogeneous risk factor preferences and dependencies. Strong evidence from epidemiological investigations and prospective researches highlighted the obesity associations both with increased incidence and poor prognosis in EAC 6,9,20. However, several lines of direct evidence assessing the roles of obesity in EAC generated inconsistent results 12, 13, 14. The “obesity paradox” phenomenon not only exists in EAC, but also can be implicated in other diseases 21, 22. However, much less is known about the underlying mechanisms behind this phenomenon. Owing to the extensive applications of the sequencing technologies to whole genomes and transcriptomes, 69 adiposity-related genes with favorable effects have been recently identified based on the large genomic database. Spurred by this paradox phenomenon and the new genetic finding, we explored the 69 OGG expression profiles and their prognostic values in EAC by the bioinformatic approach. The results showed the OGG expression levels were significantly different between normal and EAC tissues. Furthermore, the OGG changed the proportions of immune cells in the TME in patients with EAC, and have vital roles in predicting the OS. It's thus tempting to speculate the OGG are functionally involved in the incidence and survival of EAC, and they, therefore, open up a whole new range of possibilities for the prevention and treatment of EAC.

For a subset of the obese patients, the favorable effects of obesity on EAC survival, seem to be the results of individual heterogeneity and therapeutic differences. However, for a large number of the obesity patients with better outcomes, the causality failed to be explained by contingency, indicating other mechanisms uncouple adiposity from poor prognosis. Consistent with previous studies, our enrichment analysis demonstrated that the OGG may improve the prognosis in patients with EAC via the growth factor receptor pathways 23, 24. Obesity could increase the levels of various growth factors, such as vascular endothelial growth factor, hepatocyte growth factor and tumor necrosis factor, creating a chronic inflammatory milieu that supports tumorigenesis 21. Experiments *in vitro* showed obese tissues could promote cancer cells invasion and migration through transactivation of growth factor receptors 24. Although the study is still in infancy about how OGG function in EAC, the GO results gave us a hint that mitochondria-related pathways may be engaged in the crosstalk between OGG and favorable outcomes. Adipose tissues are sources of energy and endocrine cytokines, and they could activate mitochondrial fatty acid oxidation. Dysregulated mitochondria increase susceptibility to adipocytokines, which are directly involved in the metabolic regulations of the whole-body, inflammatory, and immune responses 25, 26. Therefore, impaired mitochondria in the obesity patients can predispose them to malignancy. Aggregating the 69 OGG with mitochondria emphasizes the metabolic rewire complexity, and they manifested the diversity by which obesity may regulate tumor mitochondrial activities to influence patients' survival.

The survival analysis and prognostic model proposed in this study were established according to SDEG. These genes can be roughly classified into several categories, including fat distribution (FAM13A), fatty acid oxidation (IGF2BP2), white adipose tissue browning (CSK, VEGFA), inflammation (DAGLB) and others (FAF1, ATXN2, MCM6, etc.). Among them, CSK and MCM6 are independent predictors for OS. CSK, the abbreviation of c-src tyrosine kinase, is located in chromosome position 15q24 27. It encodes the C-Src protein, which is essential for vascular smooth muscle cells and neurol differentiation 27, 28. The experiment *in vivo* by Imamoto A et al. showed the disruption of CSK genes could lead to embryonic lethality in mice through activation of c-src family kinase 28. Besides its roles in neurovascular and embryo development, the evidences of CSK in tumors are being gradually discovered as well. Early in 1992, Armstrong E et al. had drawn a link between the overexpression of CKS and the attenuated tumor metastasis ability, enforcing the notion that CSK was a potential antioncogene 29. Then, the tumor-suppressing role was further identified in Nakagawa T et al. research 30. In agreement with previous studies, our analysis also demonstrated CKS was a protective gene (HR<1) in EAC. Therefore, it may be interesting to attempt to selectively upregulate the CSK to reverse malignant biological behavior in EAC.

Cancer cell unlimited proliferation is a hallmark of EAC, and spreads indispensable effects on the malignant biological behavior. MCM6, a subunit of the mini-chromosome maintenance protein complex, maintains the functions as elements of DNA replication, genome stability, and chromatin remodeling 31, 32. The research found MCM6 was an oncogene and could promote esophageal cancer cells proliferation 33. Knockdown of MCM6 could significantly inhibit the forming of mediator of DNA-damage checkpoint 1, causing the DNA repair defects and decreased proliferation in esophageal cancer cells 33, 34. Our results were similar to their findings, unraveling the MCM6 was upregulated in EAC tissues and played an oncogenic role. Beyond these, the prognostic values of MCM6 in tumors have already been confirmed in several studies 31, 35. This was supported by Liu Z et al., who reported MCM6 was associated with poor outcomes in hepatocellular carcinoma patients (P=0.002) 31. In addition, Winnepenninckx V et al. demonstrated MCM6 independently predicted poorer survival (P=0.003) based on gene microarray analysis 35. In accordance with previously published studies, our results manifested that MCM6 was an independent risk factor for OS (HR=1.882, P=0.025). Despite we get an incomplete understanding of how the OGG drive or suppress tumors, some mechanistic clues are surfacing. For example, a growing body of researches from cell and mice-based experiments have tightly linked OGG (such as CSK and MCM6) with immunity 36, 37.

The notion that obesity modifies immune cells, mediates immune dysfunction, and promotes tumor growth is well accepted. Mounting evidence for this comes from experimental and clinical studies. Obesity directly drives adipocyte cells towards inflammatory phenotypes, releasing pro-inflammatory cytokines, such as leptin, IL-6, IL-10, TNF-α. Then, the chemotaxis of cytokines could recruit the CD4^+^ T cell, CD8^+^ T cell, macrophage M1, B cells, dendritic cells, and macrophages to infiltrate into adipocyte tissues 38, 39, 40. Our study shared similarities with these findings, revealing that the OGG significantly could influence the immune cells components (Table 2). The adipocyte tissue and immune cells synergistically resulted in the increase of steroid hormone (estrogen, androgen), proangiogenic factors (VEGF, HIF-1α), free fatty acid release, reactive oxygen species, and insulin resistance 39, 40. Under the disturbing microenvironment, seminal events that stimulate cell cancerization took place consequently, including DNA damage, neovascularization, cell invasion and migration. Indeed, immune cells contents in TME are different from those in adipocyte tissues, and it's mostly infiltrated by immunosuppressive cells, for example, macrophage M2, T cells regulatory (Tregs) 39, 41. Immune suppressors not only create favorable conditions for cancer growth, but also indirectly contribute to therapeutic resistance. Therefore, preclinical trials aimed to rescue the dysfunctional immune cells are under way now. Immunotherapy alone, or combination with other therapies (chimeric antigen receptor therapy, monoclonal antibody, and cytokine therapies), is expected to achieve satisfactory effects 42, 43, 44.

The strength of the present study is that we performed a systematic analysis about the OGG and EAC based on the national database for the first time, which provides reliable statistical evidence, and summarized the state-of-art knowledge in this field. The results are also verified in a real-world experiment. Our study, however, had some limitations that should be addressed. Firstly, the specific mechanisms of how the OGG mediate the protective survival effects and modulate the immunity are unclear. Secondly, some information in TCGA database is unavailable, for example, the chemoradiotherapy regime, which may pose significance on survival analysis and change the prognostic results. Finally, the prognostic model for OS is not tested in external cohorts, and further verification in a large-scale and multicenter clinical population is needed. Notwithstanding its limitations, this study does provide a comprehensive overview of the OGG signatures in EAC and these limitations can be solved if there are enough data in the future.

In conclusion, we identified 69 “obesity-guarding” genes signatures in patients with EAC, and they may uncouple obesity from poor survival. These genes have prognostic values in predicting the OS, and new efforts target EAC should incorporate the idea that “obesity-guarding” genes could reshape immunity.

## Supplementary Material

Supplementary tables.

## Figures and Tables

**Table 1:** Significantly different genes expression levels between normal and EAC tissues

Gene	normal	EAC	logFC	P value	FDR
FAF1	5.885	7.574	0.364	0.011	0.028
MCM6	6.163	15.897	1.367	0.001	0.001
ADAMTS9-AS2	0.447	0.093	-2.272	0.009	0.026
IGF2BP2	8.358	19.494	1.222	0.001	0.005
FAM13A	3.574	1.683	-1.086	0.000	0.003
SLC39A8	2.120	6.081	1.521	0.002	0.008
VEGFA	7.275	19.819	1.446	0.001	0.002
CENPW	3.432	14.316	2.061	0.001	0.000
DAGLB	3.505	5.157	0.557	0.004	0.017
CDC123	10.484	25.324	1.272	0.001	0.001
NT5C2	7.159	10.414	0.541	0.013	0.030
TCF7L2	9.714	16.189	0.737	0.009	0.026
NCR3LG1	2.016	4.881	1.276	0.005	0.019
HOXC6	0.443	2.031	2.196	0.001	0.002
ATXN2	4.154	5.610	0.433	0.008	0.026
CSK	15.400	23.335	0.600	0.012	0.030
TOMM40	8.938	25.297	1.501	0.001	0.001

logFC: log fold change; FDR: false discovery rate.

**Table 2:** Correlation analysis between SDEG and immune cells

Immune cell\Gene	ADAMTS9-AS2	CDC123	CENPW	CSK	DAGLB	FAM13A	HOXC6	SLC39A8	TCF7L2	TOMM40	VEGFA
Macrophages M0	0.515	0.991	0.442	0.536	0.017*	0.991	0.907	0.727	0.420	0.567	0.833
Macrophages M1	0.006*	0.232	0.371	0.083	0.407	0.046	0.102	0.049*	0.540	0.060	0.906
Dendritic cells resting	0.024*	0.118	0.632	0.022*	0.608	0.743	0.196	1.000	0.007*	0.044*	0.038*
Dendritic cells activated	0.038*	0.013*	0.675	0.159	0.617	0.051	0.318	0.450	0.223	0.003*	0.981
Mast cells resting	0.012*	0.003*	0.061	0.466	0.037*	0.322	0.337	0.924	0.247	0.001*	0.179
Mast cells activated	0.161	0.025*	0.089	0.382	0.070	0.444	0.084	0.893	0.600	0.001*	0.981
T cells follicular helper	0.641	0.035*	0.327	0.897	0.145	0.114	0.068	0.312	0.662	0.546	0.213
T cells CD4 memory resting	0.240	0.750	0.030^*^	0.916	0.815	0.825	0.815	0.642	0.393	0.222	0.833
T cells CD4 memory activated	0.952	0.458	0.895	0.972	0.476	0.531	0.943	0.039*	0.056	0.708	0.560
NK cells activated	0.099	0.273	0.033^*^	0.686	0.294	0.907	0.916	0.405	0.686	0.729	0.875
T cells regulatory (Tregs)	0.403	0.473	0.933	0.741	0.027*	0.050^*^	0.343	0.533	0.547	0.708	0.099
Plasma cells	0.249	0.212	0.710	0.218	0.140	0.455	0.048*	0.156	0.374	0.376	0.182

Table 2 summarized the SDEG correlations with immune cells. Immune cells presented in the table were significantly correlated with at least one of the genes. Other six SDEG (FAF1, IGF2BP2, CDC123, NT5C2, NCR3LG1 and ATXN2) and other 10 immune cells (B cells naive, B cells memory, T cells CD8, T cells CD4 naive, T cells gamma delta, NK cells resting, Monocytes, Macrophages M2, eosinophils and neutrophils) without significantly statistical correlation were not reported in the table.

## Abbreviations

EAC: esophageal adenocarcinoma
SEER: Surveillance Epidemiology and End Results
TME: tumor microenvironment
BMI: body mass index
OS: overall survival
GWAS: genome-wide association studies
TCGA: The Cancer Genome Atlas
SDEG: significantly differentially expressed genes
FDR: false discovery rate
OGG: “obesity-guarding” genes
GO: Gene Ontology
BP: biological process
CC: cellular component
MF: molecular function
KEGG: Kyoto Encyclopedia of Genes and Genomes
PPI: Protein-protein interaction
ROC: Receiver Operating Characteristic
AUC: area under curve
PCR: polymerase chain reaction
cDNA: complementary DNA
gDNA: genomic DNA
RT-PCR: Reverse Transcription PCR
GAPDH: glyceraldehyde-3-phosphate dehydrogenase
HR: hazard ratio
